# Formation of Silver Nanoclusters from a DNA Template Containing Ag(I)-Mediated Base Pairs

**DOI:** 10.1155/2016/7485125

**Published:** 2016-02-04

**Authors:** J. Christian Léon, Linda Stegemann, Martin Peterlechner, Stefanie Litau, Gerhard Wilde, Cristian A. Strassert, Jens Müller

**Affiliations:** ^1^Westfälische Wilhelms-Universität Münster, Institut für Anorganische und Analytische Chemie, Corrensstraße 28/30, 48149 Münster, Germany; ^2^Westfälische Wilhelms-Universität Münster, CeNTech, Physikalisches Institut, Heisenbergstraße 11, 48149 Münster, Germany; ^3^Westfälische Wilhelms-Universität Münster, Institut für Materialphysik, Wilhelm-Klemm-Straße 10, 48149 Münster, Germany

## Abstract

A series of DNA double helices containing different numbers of silver(I)-mediated base pairs involving the artificial nucleobases imidazole or 2-methylimidazole has been applied for the generation of DNA-templated silver nanoclusters. The original Ag(I)-containing nucleic acids as well as the resulting nanoclusters and nanoparticles have been characterized by means of UV/Vis spectroscopy, circular dichroism (CD) spectroscopy, fluorescence spectroscopy, and transmission electron microscopy (TEM). The results show for the first time that metal-mediated base pairs can be used for the templated growth of metal nanoclusters.

## 1. Introduction

Metal nanoclusters are a topical area of research due to their enormous applicability [1, 2]. During the past years, the use of DNA-templated nanoclusters has drawn a lot of interest [3, 4]. Depending on the experimental conditions, including the choice of appropriate oligonucleotide sequences, silver nanoclusters (AgNCs) of various sizes (and hence with diverse physical properties) can be obtained [5]. Even clusters as small as Ag_3_ or Ag_2_ have been synthesized [6]. In general, optical properties of the AgNCs appear to depend mainly on the cluster size, even though their interaction with DNA strongly affects these properties [5]. By choosing certain oligonucleotide sequences for the DNA-templated synthesis, distinct optical properties can be obtained [7–9]. First applications for DNA-templated AgNCs have been reported, too, including, for example, the detection of explosives [10]. Oligonucleotide sequences used for the templated synthesis of AgNCs often contain a large number of cytosine residues, probably as a result of the increased affinity of this canonical nucleobase for Ag(I) [11].

**Scheme 1:**
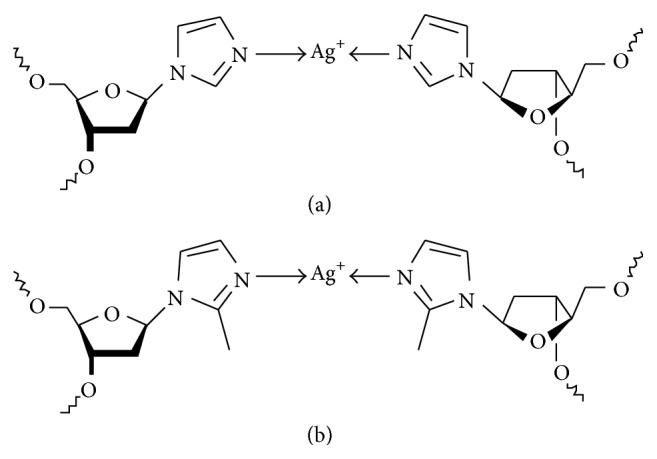
Chemical structure of the Ag(I)-mediated base pairs used in this study. (a) Imidazole–Ag(I)–imidazole; (b) 2-methylimidazole–Ag(I)–2-methylimidazole.

Within the last decade, the use of metal-mediated base pairs has been established as a versatile means for the site-specific introduction of metal-based functionality into nucleic acids [12–16]. In these base pairs, the natural nucleobases (held together by hydrogen bonds) are formally replaced by artificial ones (held together by coordinate bonds to a central metal ion). Ag(I)-mediated base pairs represent a prominent category of this novel type of base pairing. A few Ag(I)-mediated base pairs comprising entirely natural bases are known, namely, cytosine–Ag(I)–cytosine [17–19], cytosine–Ag(I)–adenine [20], and cytosine–Ag(I)–thymine [21], giving a possible rationale for the above-mentioned tendency to use cytosine-rich oligonucleotides for the templated synthesis of AgNCs. Moreover, a variety of Ag(I)-mediated base pairs with artificial nucleosides have been reported, for example, 1-deazaadenine–Ag(I)–thymine [22], pyridine–Ag(I)–pyridine [23], and base pairs with purine-derived ligands [24, 25]. Even dinuclear base pairs are known [26–31]. The most prominent and best investigated artificial Ag(I)-mediated base pairs contain imidazole nucleosides (Scheme 1) [32–37].

As was shown by means of an experimental NMR solution structure and its subsequent refinement by QM/MM methods [33, 34], the imidazole–Ag(I)–imidazole (Im–Ag–Im) base pair nicely fits into a regular B-DNA duplex without significantly distorting the nucleic acid backbone. Moreover, the affinity of an imidazole:imidazole mispair for an Ag(I) ion is about 10-fold higher than that of a cytosine:cytosine mispair [37]. Hence, we set out to investigate the use of Im–Ag–Im base pairs for the generation of DNA-templated AgNCs.

## 2. Materials and Methods

The artificial nucleosides were synthesized according to literature procedures [33, 35]. Phosphoramidites of the canonical nucleosides were purchased from Glen Research. Oligonucleotide synthesis and purification were performed as previously reported [26]. Oligonucleotide concentrations were determined based on their absorbance. For the artificial nucleosides, a molar extinction coefficient *ε*_260_ = 0.1 cm^2^ mmol^−1^ was used. The desalted oligonucleotides were characterized by MALDI-TOF mass spectrometry (ODN1: calcd. for [M + H]^+^: 6498 Da, found: 6498 Da; ODN2: calcd. for [M + H]^+^: 6464 Da, found: 6465 Da; ODN3: calcd. for [M + H]^+^: 6744 Da, found: 6745 Da; ODN4: calcd. for [M + H]^+^: 6710 Da, found: 6712 Da; ODN5: calcd. for [M + H]^+^: 6554 Da, found: 6555 Da; ODN6: calcd. for [M + H]^+^: 6520 Da, found: 6521 Da; ODN7: calcd. for [M + H]^+^: 6814 Da, found: 6814 Da; ODN8: calcd. for [M + H]^+^: 6780 Da, found: 6780 Da; ODN9: calcd. for [M + H]^+^: 5514 Da, found: 5512 Da; ODN10: calcd. for [M + H]^+^: 5481 Da, found: 5477 Da). MALDI-TOF mass spectra were recorded on a Bruker Reflex IV instrument using a 3-hydroxypicolinic acid/ammonium citrate matrix. Temperature-dependent UV measurements were performed on a UV spectrometer CARY 100 Bio instrument. UV melting curves were recorded from 10 to 80°C using solutions containing 2 *μ*M oligonucleotide duplex, 150 mM NaClO_4_, 5 mM MOPS (pH 6.8), and an appropriate amount of AgNO_3_ with a heating/cooling rate of 1°C min^−1^ and a data interval of 0.5°C. Absorbance was normalized according to *A*_norm_ = (*A* − *A*_min_)/(*A*_max_ − *A*_min_) at 260 nm. Melting temperatures were determined from the maxima of the first derivatives of the melting curves. Reduction of the metal-mediated base pairs was performed at ambient temperature by adding a freshly prepared aqueous solution of NaBH_4_ to a solution containing 40 *μ*M oligonucleotide duplex (in 150 mM NaClO_4_, one equivalent of AgNO_3_, and 5 mM MOPS (pH 6.8)) that had previously been heated to 65°C and then cooled to 10°C at a rate of 1°C min^−1^. In the case of duplex** V** and the nucleic-acid-free reference measurement, 160 *μ*M AgNO_3_ was used in the same aqueous buffer. UV/Vis spectra of the reduced solutions were recorded on a Nanodrop 2000c instrument. They were normalized with respect to a 1 cm path length (assuming *ε*_260_ = 332 cm^2^ *μ*mol^−1^ for oligonucleotide duplexes** I**–**IV** and *ε*_260_ = 424 cm^2^ *μ*mol^−1^ for duplex** V**) and smoothed. For the nucleic-acid-free solution, a scaling factor of 18 (similar to the ones calculated for duplexes** I**,** III**, and** V** with the same Ag(I) concentration as the nucleic-acid-free solution) was applied. CD spectra were recorded at 10°C on a JASCO J-815 spectropolarimeter, and a manual baseline correction was applied. The TEM measurements were carried out on a Libra 200 FE TEM (Zeiss) equipped with a field emission gun operating at 200 kV, an in-column energy filter, a Noran energy dispersive X-ray (EDX) detector, and a Gatan US4000 CCD. Conventional TEM data were recorded using an energy filter window size of 30 eV symmetrically around the zero loss. TEM samples were obtained using Cu-grids with a holey carbon film by slewing the grids in the solutions. Steady-state emission spectra were recorded on a FluoTime300 spectrometer from PicoQuant equipped with a 300 W ozone-free Xe lamp (250–900 nm), an excitation monochromator (Czerny-Turner 2.7 nm mm^−1^ dispersion, 1200 grooves mm^−1^, blazed at 300 nm), two emission monochromators (Czerny-Turner, selectable gratings blazed at 500 nm with 2.7 nm mm^−1^ dispersion and 1200 grooves mm^−1^, or blazed at 1250 nm with 5.4 nm mm^−1^ dispersion and 600 grooves mm^−1^), and a PMA Hybrid 40 (transit time spread FWHM <120 ps, 300–720 nm). Emission spectra were corrected for source intensity (lamp and grating) by standard correction curves.

## 3. Results and Discussion

### 3.1. Characterization of the Ag(I)-Binding Behavior of the Oligonucleotides

Five slightly different nucleic acid duplexes were investigated with respect to their capability to act as templates for the formation of AgNCs.  Table 1 gives an overview of the sequences. Duplexes** I** and** III** comprise four designated Ag(I)-binding sites, duplexes** II** and** IV** five designed Ag(I)-binding sites. The artificial nucleobases used to generate the binding sites are imidazole (duplexes** I** and** II**) and 2-methylimidazole (duplexes** III** and** IV**). The latter constitutes a 2nd generation imidazole-type nucleobase with an increased thermal stability of the resulting Ag(I)-mediated base pair, in which for steric reasons the central Ag(I) ion is expected to have a smaller solvent accessibility [35]. Duplex** V** serves are a reference duplex without any artificial nucleobases.

To ensure that indeed distinct Ag(I)-mediated base pairs are formed, melting curves of the duplexes were determined by temperature-dependent UV spectroscopy in the presence of increasing amounts of Ag(I). During the following discussion, one equivalent of Ag(I) is defined as the amount of Ag(I) required for the formation of all possible Ag(I)-mediated base pairs. Hence, for duplexes** I** and** III** one equivalent equals four Ag(I) ions, whereas for duplexes** II** and** IV** one equivalent equals five Ag(I) ions. This definition allows the discussion of the different duplexes irrespective of their absolute Ag(I) content.

**Figure 1:**
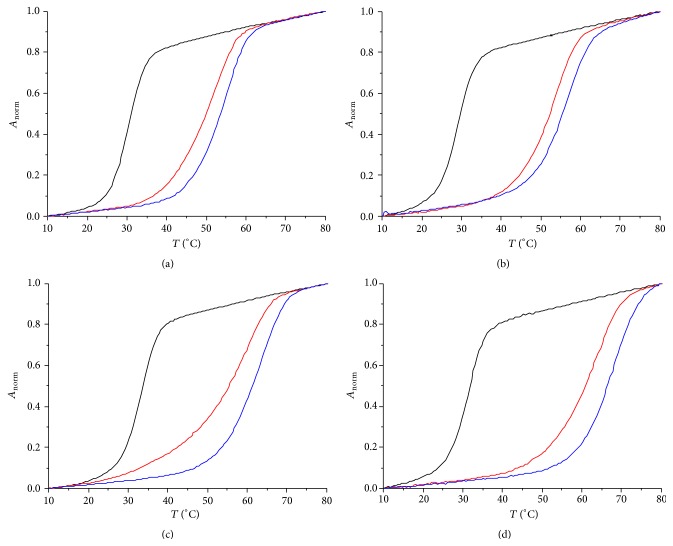
Melting curves of duplexes** I**–**IV** in the presence of various equivalents of Ag(I) (black: no Ag(I); red: 1 equiv. of Ag(I); blue: 1.5 equiv. of Ag(I)): (a) duplex** I**; (b) duplex** II**; (c) duplex** III**; (d) duplex** IV**.

As can be seen from the melting curves shown in Figure 1, the addition of Ag(I) leads to a large thermal stabilization. Interestingly, the addition of excess Ag(I) results in a rather small additional increase of the melting temperature *T*_*m*_. This behavior (large increase in *T*_*m*_ upon the addition of one equivalent of Ag(I), much smaller increase thereafter) is typical for the formation of metal-mediated base pairs [12]. It indicates that the artificial mispairs indeed represent the highest-affinity binding sites for Ag(I). This is confirmed by a reference measurement using duplex** V** without any artificial nucleosides. The addition of Ag(I) to duplex** V** leads to an increase in *T*_*m*_ of a little more than 1°C per Ag(I) only (Δ*T*_*m*_ = 6°C for four Ag(I)). For duplexes** I**–**IV**, additional Ag(I) probably binds nonspecifically to the negatively charged nucleic acid backbone [38] or to vacant sites on the canonical nucleobases [39].  Table 2 lists the melting curves of the duplexes in the absence and presence of one equivalent of Ag(I), that is, with and without Ag(I)-mediated base pairs. As expected, the use of 2-methylimidazole leads to the formation of thermally more stable Ag(I)-mediated base pairs. The average stabilization per individual Ag(I)-mediated base pairs amounts to 5-6°C.

**Figure 2:**
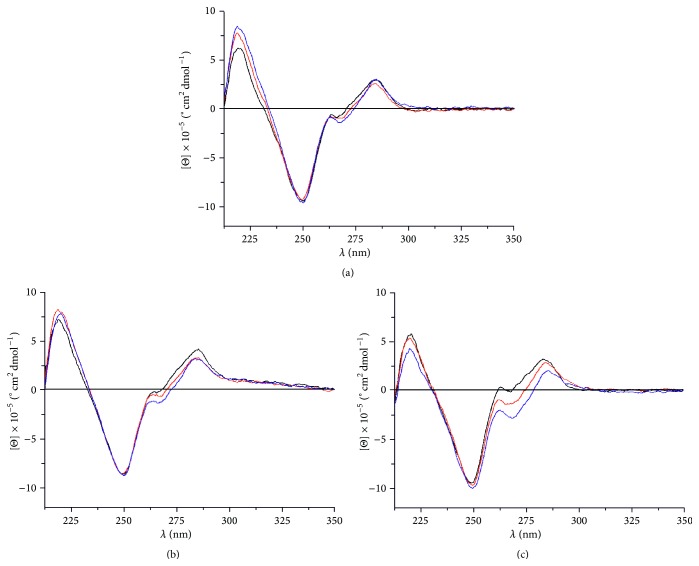
CD spectra of (a) duplex** I**, (b) duplex** II**, and (c) duplex** V** in the absence or presence of Ag(I) (black: no Ag(I); red: 1 equiv. of Ag(I); blue: 1.5 equiv. of Ag(I)). The CD spectra of duplexes** III** and** IV** including 2-methylimidazole rather than imidazole are essentially the same [35, 37] and are therefore not shown. In (c), a different color code applies (red: 4 Ag(I) per duplex; blue: 6 Ag(I) per duplex).

Circular dichroism (CD) spectroscopy was performed to elucidate the conformations of the Ag(I)-free and the Ag(I)-containing nucleic acid duplexes. As can be seen from the CD spectra shown in Figure 2, the addition of Ag(I) does not induce significant conformational changes to the duplexes. Minor changes are seen in the CD spectrum of duplex** II**, which is not unexpected, because it contains the largest number of mispairs among the investigated duplexes that become metal-mediated base pairs in the presence of Ag(I). Importantly, the CD spectrum of the native DNA duplex** V** in the presence of increasing amounts of Ag(I) shows a decrease in molar ellipticity at around 270 nm. A similar change becomes evident in the CD spectra of duplexes** I** and** II** only if more than one equivalent of Ag(I) is present, thereby confirming that the imidazole-based mispairs represent the highest-affinity binding sites for Ag(I). In general, the CD spectra resemble those of duplexes rich in adenine:thymine base pairs consisting of one purine-rich and one pyrimidine-rich strand [40].

### 3.2. Spectroscopic Properties of the DNA-Templated Ag Nanoclusters

Reduction of the Ag(I) ions to give AgNCs was achieved by the addition of an excess of a freshly prepared aqueous solution of NaBH_4_. Contrary to the experiments described in Section 3.1, duplex concentrations of 40 *μ*M were used. A uniform formation of oligonucleotide duplexes with Ag(I)-mediated base pairs was achieved by heating buffered aqueous solutions of the duplexes to 65°C and slowly cooling them to 10°C in the presence of one equivalent of AgNO_3_.

**Figure 3:**
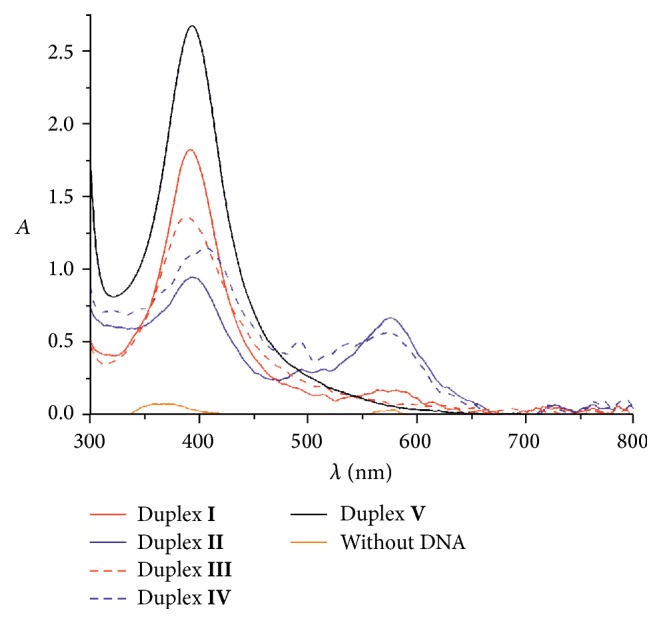
Section of the UV/Vis spectra of duplexes** I**–**V** after the reduction by NaBH_4_. In the absence of DNA, practically no absorbance is detected.

Upon reduction, the colorless oligonucleotide solutions turned yellowish brown. The UV/Vis spectra clearly indicate the presence of new absorbance maxima in the visible range (Figure 3). In particular, a new peak at ~395 nm is observed for all four imidazole-comprising duplexes as well as for the native DNA duplex. An additional peak at ~575 nm can be found in the spectra of duplexes** II** and** IV** and to a smaller extent also in the spectrum of duplex** I**. The spectra of duplexes** II** and** IV** also feature smaller peaks around ~495 nm.  Table 3 lists the UV/Vis spectroscopic data of duplexes** I**–**V** after the addition of NaBH_4_. The peak around 395–405 nm indicates the formation of silver nanoparticles with a diameter of 10–14 nm [41]. With time, this peak becomes broader and red-shifted, indicating a further aggregation of the nanoparticles [41]. In contrast, the additional peak at ~575 nm has previously been associated with the formation of nanoclusters [42] and hence indicates that the anticipated AgNCs were formed as well. It is interesting to note that the reduction of AgNO_3_ by NaBH_4_ in the absence of any DNA does not lead to a colored solution (Figure 3, orange spectrum), indicating the absence of small silver nanoparticles or nanoclusters. Moreover, the reduction of AgNO_3_ in the presence of the native duplex** V** does not lead to species with an absorbance maximum above 500 nm.

**Figure 4:**
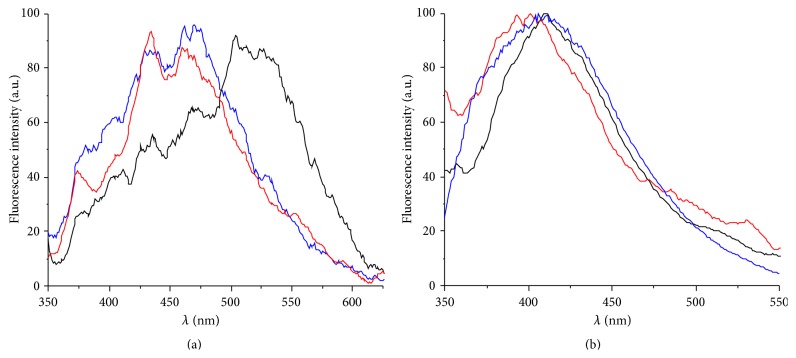
Fluorescence spectra of (a) duplex** I** and (b) duplex** II** upon excitation at 330 nm (blue: in the absence of Ag(I); black: in the presence of 1 equiv. of Ag(I); red: after reduction by NaBH_4_). The weak intensity indicates that the samples are essentially nonfluorescent.

As AgNCs are well-known for their fluorescent properties [1], the reduced nucleic acid solutions of duplexes** I** and** II** were subjected to fluorescence spectroscopic characterization. As indicated by Figure 4, no significant fluorescence signal was detected upon the reduction of the Ag(I)-containing oligonucleotide duplexes. The marginal fluorescence around 400–550 nm observed both prior to and after the reduction can most likely be attributed to the intrinsic fluorescence of DNA, as was shown by recording fluorescence spectra also in the absence of any Ag(I) and of any AgNCs. The lack of fluorescence may be explained by the use of a double-stranded DNA template, as duplex DNA has previously been shown to produce AgNCs with negligible visible fluorescence [7]. It has been suggested that this may be due to electronic configurations without excitations in the visible range and/or excitations that decay rapidly in a nonradiative manner [7].

### 3.3. TEM Measurements

**Figure 5:**
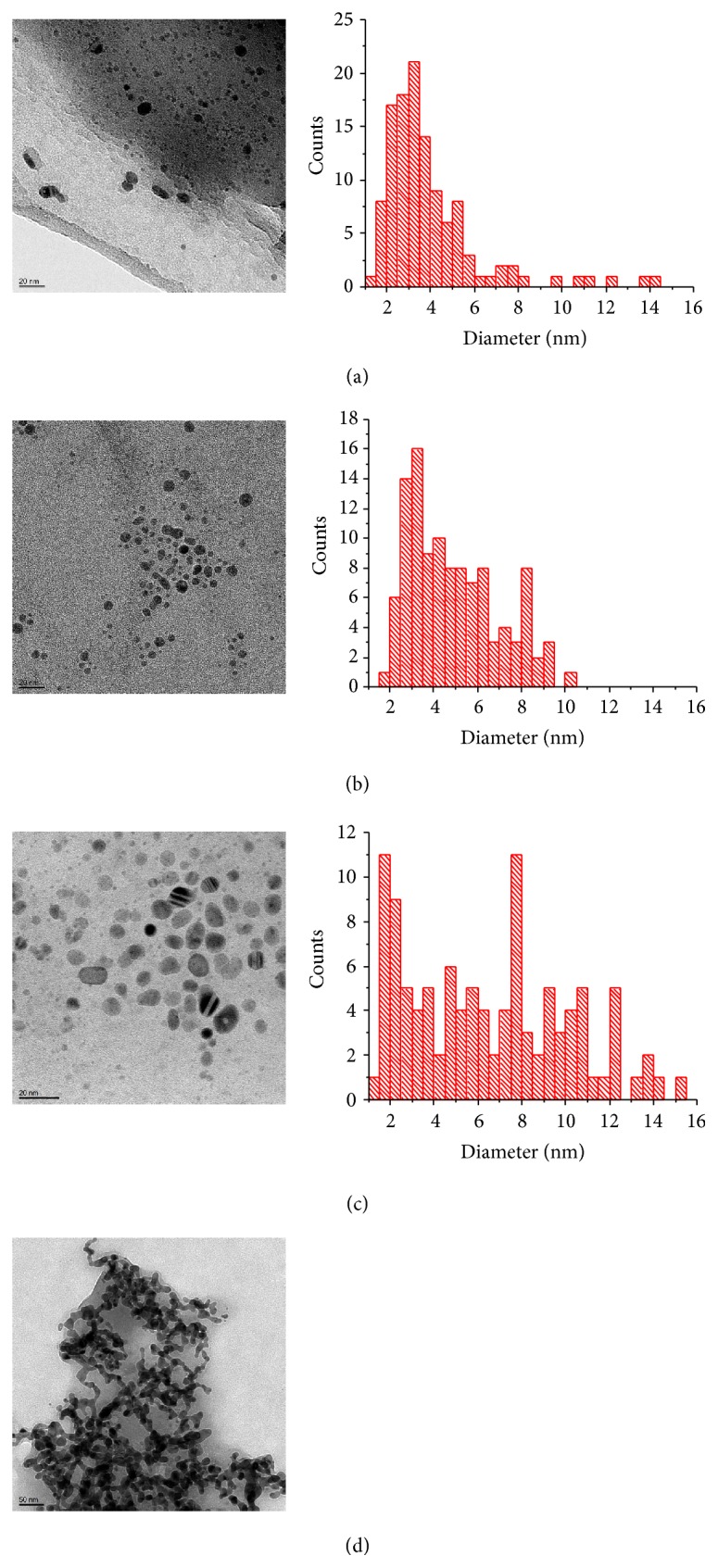
TEM bright-field images of silver nanoclusters and nanoparticles on a carbon film. Solutions of (a) duplex** I**, (b) duplex** II**, (c) duplex** V**, and (d) without nucleic acid in the presence of AgNO_3_ after the reduction with NaBH_4_ led to the shown images and corresponding histograms of the size distribution. Please note the different scale bars in (c) and (d).

To investigate whether, despite the lack of significant fluorescence, AgNCs have been obtained, TEM measurements were performed for the reduced solutions of duplexes** I**,** II**, and** V**. The resulting images confirm that nanoparticles (>10 nm diameter) and nanoclusters (<10 nm diameter) are formed. EDX spectroscopy confirms the particles to be composed of silver. In agreement with that, diffraction experiments combined with dark-/bright-field imaging shows single crystalline silver particles.  Figure 5 shows selected TEM images devoid of larger aggregates. As can be seen, the particle diameter in these sections is mostly in the order of 2 nm–10 nm. The particles are basically spherical. The existence of additional nanoclusters smaller than 2 nm cannot be ruled out, as these are below the resolution of the present TEM experiments using holey carbon grids. The average size of the nanoclusters obtained from duplex** II** (with 2-methylimidazole) shown in Figure 5 is within standard error identical (*d* = 5 ± 2 nm, *N* = 111) to the size of those obtained from duplex** I** (with imidazole) (*d* = 4 ± 2 nm, *N* = 118). Hence, there seems to be no simple correlation between the number of Ag(I)-mediated base pairs and the size distribution of the resulting AgNCs. For the native DNA duplex** V**, a much wider size distribution was observed (Figure 5(c), *d* = 6 ± 4 nm, *N* = 107). In general, the silver nanoparticles tend to be a bit larger than those obtained from DNA with Ag(I)-mediated base pairs. Finally, the reduction of an identical concentration of Ag(I) in the absence of DNA led to a completely different appearance of the resulting silver (Figure 5(d)). Individual spherical nanoparticles or nanoclusters were either not formed or not stable in this case, as only aggregates were observed. Hence, the TEM measurements show that the nucleic acids with Ag(I)-mediated base pairs give the smallest AgNCs with the most narrow size distribution.

## 4. Conclusions

This study shows for the first time that nucleic acids with artificial Ag(I)-mediated base pairs can be applied for the generation of silver nanoclusters. Formation of the clusters was confirmed by TEM. Despite the fact that the nucleic acids contain a discrete number of Ag(I) ions per duplex, the AgNCs were not found to be monodisperse. Nonetheless, a much more narrow size distribution was observed compared with an entirely natural DNA duplex template, indicating the relevance of the Ag(I)-mediated base pairs for cluster formation. Moreover, the nanoclusters formed from DNA with Ag(I)-mediated base pairs have different spectroscopic properties compared with those formed from native DNA in the presence of AgNO_3_, as evident from the absorbance maxima in their UV/Vis spectra. The initially formed small nanoclusters have a tendency to aggregate in the case of duplexes** I**–**IV**. Such a time-dependent growth of nanoparticles was followed UV/Vis-spectroscopically. Considering that numerous metal-mediated base pairs have been established for a range of different metal ions, this study opens a new and promising avenue towards the formation of nanoclusters of different metals. Depending on the identity of the metal ion included in the metal-mediated base pair, different nanoclusters with distinct physical properties should be obtainable.

## Figures and Tables

**Table 1:** DNA oligonucleotide duplexes and sequences investigated in this study.

Duplex	Sequence^a^	Sequence number
**I**	5′-d(GTT TGT TTG **XXX** **X** TG TTT TTT T)	ODN1
	3′-d(CAA ACA AAC **XXX** **X** AC AAA AAA A)	ODN2

**II**	5′-d(GTT TGT TTG **XXX** **XX** T GTT TTT TT)	ODN3
	3′-d(CAA ACA AAC **XXX** **XX** A CAA AAA AA)	ODN4

**III**	5′-d(GTT TGT TTG **YYY** **Y** TG TTT TTT T)	ODN5
	3′-d(CAA ACA AAC **YYY** **Y** AC AAA AAA A)	ODN6

**IV**	5′-d(GTT TGT TTG **YYY** **YY** T GTT TTT TT)	ODN7
	3′-d(CAA ACA AAC **YYY** **YY** A CAA AAA AA)	ODN8

**V**	5′-d(GTT TGT TTG TGT TTT GTT TTT TT)	ODN9
	3′-d(CAA ACA AAC ACA AAA CAA AAA AA)	ODN10

^a^ **X** = imidazole nucleoside, **Y** = 2-methylimidazole nucleoside.

**Table 2:** Melting temperatures *T*_*m*_/°C of the duplexes in the absence and presence of Ag(I)^a^.

	0 equivalent of Ag(I)	1 equivalent of Ag(I)	Δ*T*_*m*_ (0 → 1 equivalent of Ag(I))
Duplex **I**	30.6	51.1	20.5
Duplex **II**	29.5	52.2	22.7
Duplex **III**	32.8	60.8	28.0
Duplex **IV**	30.7	60.7	30.0
Duplex **V**	52.4	58.4^b^	6.0^b^

^a^Conditions: 2 *μ*M duplex, 150 mM NaClO_4_, and 5 mM MOPS (pH 6.8). ^b^Four Ag(I) were added to the duplex.

**Table 3:** UV/Vis spectroscopic data of silver nanoparticles and nanoclusters formed upon reduction of Ag(I)-containing duplexes **I**–**V** ^a^.

	Duplex **I**	Duplex **II**	Duplex **III**	Duplex **IV**	Duplex **V**
*λ*_max_/nm	393, 582	394, 495, 577	389	406, 494, 571	394

^a^Conditions: 40 *μ*M duplex, 1 equivalent AgNO_3_ (duplex **V**: 4 AgNO_3_ per duplex), 150 mM NaClO_4_, 5 mM MOPS (pH 6.8), and excess NaBH_4_ solution.
