# Corrigendum to “Formation of Silver Nanoclusters from a DNA Template Containing Ag(I)-Mediated Base Pairs”

**DOI:** 10.1155/2018/7897953

**Published:** 2018-06-21

**Authors:** J. Christian Léon, Linda Stegemann, Martin Peterlechner, Stefanie Litau, Gerhard Wilde, Cristian A. Strassert, Jens Müller

**Affiliations:** ^1^Institut für Anorganische und Analytische Chemie, Westfälische Wilhelms-Universität Münster, Corrensstraße 28/30, 48149 Münster, Germany; ^2^CeNTech, Physikalisches Institut, Westfälische Wilhelms-Universität Münster, Heisenbergstraße 11, 48149 Münster, Germany; ^3^Institut für Materialphysik, Westfälische Wilhelms-Universität Münster, Wilhelm-Klemm-Straße 10, 48149 Münster, Germany

In the article titled “Formation of Silver Nanoclusters from a DNA Template Containing Ag(I)-Mediated Base Pairs” [1], there was an error in the sequences of duplex V in Table 1, which should be corrected as follows:

## Figures and Tables

**Table 1:** DNA oligonucleotide duplexes and sequences investigated in this study.

Duplex	Sequence^a^	Sequence number
I	5′-d(GTT TGT TTG XXX XTG TTT TTT T)	ODN1
	3′-d(CAA ACA AAC XXX XAC AAA AAA A)	ODN2
II	5′-d(GTT TGT TTG XXX XXT GTT TTT TT)	ODN3
	3′-d(CAA ACA AAC XXX XXA CAA AAA AA)	ODN4
III	5′-d(GTT TGT TTG YYY YTG TTT TTT T)	ODN5
	3′-d(CAA ACA AAC YYY YAC AAA AAA A)	ODN6
IV	5′-d(GTT TGT TTG YYY YYT GTT TTT TT)	ODN7
	3′-d(CAA ACA AAC YYY YYA CAA AAA AA)	ODN8
V	5′-d(GTT TGT TTG TGT TTT TTT)	ODN9
	3′-d(CAA ACA AAC ACA AAA AAA)	ODN10

^a^X = imidazole nucleoside; Y = 2-methylimidazole nucleoside.
